# Distributed Bees Algorithm Parameters Optimization for a Cost Efficient Target Allocation in Swarms of Robots

**DOI:** 10.3390/s111110880

**Published:** 2011-11-21

**Authors:** Aleksandar Jevtić, Álvaro Gutiérrez

**Affiliations:** ETSI Telecomunicación, Universidad Politécnica de Madrid, Av. Complutense 30, 28040 Madrid, Spain; E-Mail: aguti@etsit.upm.es

**Keywords:** swarm robotics, multi-agent systems, cooperative sensors, distributed task allocation, parameter optimization, genetic algorithms

## Abstract

Swarms of robots can use their sensing abilities to explore unknown environments and deploy on sites of interest. In this task, a large number of robots is more effective than a single unit because of their ability to quickly cover the area. However, the coordination of large teams of robots is not an easy problem, especially when the resources for the deployment are limited. In this paper, the Distributed Bees Algorithm (DBA), previously proposed by the authors, is optimized and applied to distributed target allocation in swarms of robots. Improved target allocation in terms of deployment cost efficiency is achieved through optimization of the DBA’s control parameters by means of a Genetic Algorithm. Experimental results show that with the optimized set of parameters, the deployment cost measured as the average distance traveled by the robots is reduced. The cost-efficient deployment is in some cases achieved at the expense of increased robots’ distribution error. Nevertheless, the proposed approach allows the swarm to adapt to the operating conditions when available resources are scarce.

## 1. Introduction

Distributed sensor networks can be used to gather information and create knowledge about an unknown environment. In applications that require area coverage, multi-robot systems with their sensing capabilities have an advantage over a single robot unit because of their ability to quickly deploy within a larger area. Some of the possible applications include planetary exploration, urban search and rescue, monitoring, surveillance, cleaning, maintenance, and so forth. In order to efficiently perform their tasks, robots require a high level of autonomy and cooperation.

Even though cheap robot hardware has become widely accessible on the market, application of multi-robot systems in our everyday lives is limited. Nevertheless, due to the potential that this field has, great efforts have been made by various research groups to investigate the algorithms for coordination and control of multi-robot systems consisting of large number of units. In order to unify the research under a single framework, some researchers have proposed different multi-robot system taxonomies. Dudek *et al*. [1] proposed a taxonomy that categorizes the existing multi-robot systems along various axes, including size (number of robots), team organization (e.g., centralized *vs*. distributed), communication topology (e.g., broadcast *vs*. unicast), and team composition (e.g., homogeneous *vs*. heterogeneous). Rather than architectures, Gerkey and Matarić [2] categorized the underlying coordination problems with a focus on multi-robot task allocation (MRTA). These authors distinguished: single-task (ST) and multi-task (MT) robots, single-robot (SR) and multi-robot (MR) tasks, and instantaneous (IA) and time-extended (TA) assignment.

When dealing with a large number of robots, distributed coordination and decentralized communication can acquire great benefits for the overall system’s performance. A system consisting of a large number of autonomous robots that directly or indirectly (via environment) communicate with one another is referred to as swarm [3]. The advantages of the decentralized over a more traditional centralized approach can be significant as the former usually provides higher autonomy, adaptability, scalability, and robustness of the whole system [4–8]. In order to develop adequate coordination models for robot swarms, many researchers have sought inspiration in natural systems, such as ant and bee colonies, bird flocks or fish schools [9–12]. Still, criteria for robot swarms remains efficiency and cost, while the biological plausibility often serves only as an initial idea.

In this paper, the optimized Distributed Bees Algorithm (DBA) is applied to distributed target allocation in a swarm of robots. The DBA was previously proposed and validated by the authors through a set of experiments with physical robots [13]. A detailed comparison of the DBA with the state of the art algorithms for task allocation, and the analysis of the algorithm’s scalability, are given in [14]. The DBA introduces a set of control parameters that adapt swarm’s behavior with respect to robots’ distribution error and deployment cost. In this work, these parameters are optimized for an improved swarm’s performance in terms of deployment cost measured as the average distance traveled by the robots in the deployment phase. By changing the values of the DBA’s control parameters, the targets’ allocation patterns are modified. The control parameters’ values are optimized by means of a Genetic Algorithm [15]. GAs have proven to be powerful optimization tools. These are population-based algorithms, where creating a population of solutions (genes) makes less probable getting stuck in a local optimum.

The remainder of this paper is organized as follows. Section 2 defines the problem statement and presents a description of the DBA. Also, in this section, the role of the algorithm’s control parameters is elaborated. Section 3 describes the simulator used for experiments and proposes the experimental setup. In Section 4 experimental results are presented and discussed. Finally, in Section 5 conclusions are made.

## 2. Multi-Robot Target Allocation

### 2.1. Scenario and Problem Statement

Based on Dudek’s taxonomy [1], the proposed multi-robot system can be categorized as homogeneous and distributed, using broadcast communication. The problem addressed in this paper is for single-task robots, multi-robot tasks and instantaneous assignment (ST-MR-IA) [2]. The task (*i.e.*, target) allocation scenario is placed in a 2-dimensional robot arena with a preset number of targets that could be of same or different importance. A finite number of robots are allowed to be allocated to any target, still each robot can only be allocated to one target at any given time. Targets have associated quality values and have their own location coordinates. The quality of a target is an application-specific scalar value that may represent target’s priority or complexity, where a higher value requires a higher number of allocated robots. The medium by which these values are obtained is not considered in this paper.

The proposed scenario is presented under the following assumptions:

- All the targets are made available to all the robots. This is done by setting a broadcast communication range of the robots to cover the entire arena.
- Robots take decision once a predefined number of targets in the arena is found. The robots that found a target are automatically allocated to that target.
- Reallocation to another target is not allowed.

These assumptions are taken for simplicity; otherwise, it would be difficult to analyze the performance of the system due to the unpredictability of the robots’ distribution prior to target allocation. It is important to mention that the entire swarm is involved in the search for targets. The experimental setup has a limitation that the robots wait for a preset number of targets to be found in order to allocate. This value can be altered or set as a variable, but that is not considered in this study and remains to be a part of future work. Even though the broadcast communication represents a centralized solution, the decision making is executed by the robots in a distributed manner, which is an inherent characteristic of swarms in nature.

The Multi-Robot Target Allocation problem can be described as follows. Consider a population of *N* robots to be allocated among *M* targets. Let *Q* ∈ {*q*_1_, . . ., *q_M_*} denote the set of normalized qualities of all available targets. We denote the number of robots on the target *i* ∈ {1, . . ., *M*} by *n_i_*, a nonnegative integer. The population fraction allocated to target *i* is *f_i_* = *n_i_*/*N*, which represents the target’s relative frequency, and the vector of population fraction is **f** = [*f*_1_, . . ., *f_M_*]*^T^*. The expected distribution is the set of desired population fractions on each target, $f^{d}={\left[f_{1}^{d},\ldots ,f_{M}^{d}\right]}^{T}$, where $f_{i}^{d}=q_{i}$. The usage of fractions rather than integers is practical for scaling, but it also introduces a distribution error as the fractions can take only certain values that are defined by the swarm size.

### 2.2. Distributed Bees Algorithm

The DBA [14] was applied to multi-robot target allocation in the proposed scenario. The robots start a search for the targets from their randomly chosen initial locations in the arena. When a robot finds a target, it broadcasts the message containing the target quality. When another robot receives information about the predefined number of targets, it calculates the utilities with respect to those targets. The utility depends on the target’s quality value and the related deployment cost measured as the robot’s distance from the target. The distance to the target is obtained thanks to a local, distributed and situated communication system [16,17]. When a robot broadcast information about the target, a receiver robot obtains the information transmitted together with the range (distance) and bearing (orientation) to the emitter robot. Therefore, the robot is able to calculate the distance and orientate to the emitting robot. The main concepts behind the implementation of the DBA are presented hereafter.

The cost of a target *i* for robot *k* is calculated as the Euclidean distance, $d_{i}^{k}$, between the robot and the target in a two-dimensional arena. However, the target’s visibility is defined as the reciprocal value of the distance:

(1) $$\eta_{i}^{k}=\frac{1}{d_{i}^{k}}.$$

The target’s quality is a scalar value that represents its priority or complexity. Normalized qualities are calculated as fractions of the sum of qualities of all available targets:

(2) $$q_{i}=\frac{Q_{i}}{\sum_{j=1}^{M}Q_{j}}.$$

where *Q_i_* is a quality of the target *i*. In real-world scenarios, the quality of a region of interest is an estimated value that results from sensor readings or a previously acquired knowledge.

The utility of a robot depends on both visibility and quality of the chosen target. The utility is defined as a probability that the robot *k* is allocated to the target *i*, and it is calculated as follows:

(3) $$p_{i}^{k}=\frac{q_{i}^{\alpha }\eta_{i}^{\beta }}{\sum_{j=1}^{M}q_{j}^{\alpha }\eta_{j}^{\beta }}$$

where *α* and *β* are control parameters that allow biasing of the decision-making mechanism towards the quality of the solution or its cost, respectively. (*α*, *β* > 0; *α*, *β* ∈ ℜ.) The GA-based method applied to optimize these parameters is introduced in Section 3.1. From Equation (3), it is easy to show that:

(4) $$\sum_{i=1}^{M}p_{i}^{k}=1.$$

The underlying decision-making mechanism of the DBA adopts the roulette rule, also known as the wheel-selection rule. That is, every target has an associated probability with which it is chosen from a set of available targets. Once all the probabilities are calculated (see Equation (3)), the robot chooses a target by “spinning the wheel”. A comparison of the DBA with the state-of-the-art task allocation algorithms was given in [14]. This paper is an extension of that work, and it focuses on the improvement of the DBA through optimization of its control parameters.

## 3. Experimental Evaluation

### 3.1. Genetic Algorithm

In the DBA optimization, two control parameters have been taken into account. The parameters *α* and *β* define how targets’ distances (*i.e.*, visibility) and quality values affect the robots’ distribution in the arena. The influence of the parameters on a target allocation probability is exponential (see Equation (3)). Hence, a small change in their values can result in very different robots’ distribution patterns, and a larger distribution error. Moreover, considering that a large number of agents can be found in a swarm, increase in the deployment cost can be very significant. Therefore, even though a simple sampling of the solution space would be less computationally demanding, in order to obtain a high accuracy and considering that the parameters optimization is performed offline, a Genetic Algorithm [15] was used. In order to limit the complexity of the exploration process, the following range of possible values was defined for both parameters: *α*, *β* ∈ [0, 5]. Initially, a population of 30 random genotypes was created, in which values are drawn from uniform distributions in the respective ranges of the parameters. The genetic algorithm was run for 1, 000 generations, during which new generations of genotypes were bred. The genetic algorithm loop consists of the evaluation, the selection and the reproduction of the genotypes.

In order to evaluate the fitness of a given genotype, the controller of 40 simulated robots was parameterized with the values of *α* and *β* encoded in the genotype. The total number of *R* = 50 simulated experiments were run with different initial conditions. The experiments duration was set to *T* = 100 s. The fitness function *F*(*g*), of the evaluated genotype *g*, is computed in Equation (5) as an indicator of the swarm’s ability to allocate the robots according to the targets’ quality distribution (*q_i_*) and visibility (*η_i_*). The fitness *F* is defined as follows:

(5) $$F=\frac{1}{\mathrm{MAE}\cdot \bar{d}}$$

where *MAE* is the mean absolute distribution error and *d̄* is the average distance traversed by all the robots.

Generations following the first one are produced by a combination of selection with elitism, recombination and mutation. For each new generation, the two highest scoring individuals (“the elite”) from the previous generation are retained unchanged. The remainder of the new population is generated by fitness-proportional selection (also known as roulette wheel selection) from the individuals of the old population. Mutation entails that a random Gaussian offset is applied to each real-valued vector component encoded in the genotype (except the elite), with a probability of 0.5. The mean of the Gaussian is *μ* = 0, and its standard deviation is *σ* = 0.1. During evolution, all vector component values are constrained to remain within the range [0,1]. Once the new population has been created, the genotype parameters are linearly mapped to produce network parameters with the aforementioned ranges (*α*, *β* ∈ [0, 5]).

### 3.2. Simulator

**Figure 1.**
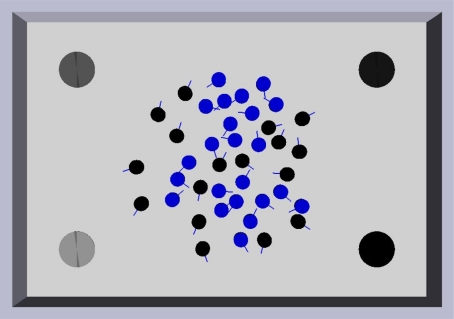
Simulator screenshot. Experimental setup included 40 robots engaged in search for 4 targets of different qualities represented by different grey-level intensity. Robots are programmed for obstacle avoidance; when robot detects an obstacle its color changes from black to blue to mark its new state. Once the robot has taken a new direction, its color goes back to black.

Our simulation platform is a fast, specialized multi-robot simulator for the e-puck robot [18] described in [19]. It is a simple and effective simulator implementing 2D kinematics. A screenshot of the simulator is shown in Figure 1. In the simulator, the e-puck is modeled as a cylindrical body of 3.5 cm in radius that holds 8 infrared (IR) proximity sensors distributed around the body, 3 ground sensors on the lower-front part of the body and a range and bearing communication sensor. IR proximity sensors have a range of 5 cm, while the communication range of the E-puck Range & Bearing module [16,17] has been set to cover the whole arena. For the three types of sensors, real robot measurements were sampled and the data was mapped into the simulator. Furthermore, uniformly distributed noise was added to the samples in order to effectively simulate different sensors; ±20% noise is added to the IR sensors and ±30% to the ground sensors. In the range and bearing sensor, noise is added to the range (±2.5 cm) and bearing (±20°) values. A differential drive system made up of two wheels is fixed to the body of the simulated robot. At each time step (100 ms), the robot senses the environment and actuates. The robot speed has been limited to 6 cm/s when moving straight and 3 cm/s when turning.

### 3.3. Experiments

Four different experimental setups have been proposed. The Experimental Setup 1 is planned to demonstrate how a change in values of the DBA’s control parameters *α* and *β* affects the swarm’s behavior. The scenario-specific parameters used in this experiment are shown in Table 1.

The experimental setup 2, 3, and 4 compare the swarm’s performance for the new and the initially-used set of *α* and *β* values, *α* = *β* = 1 [14]. These sets are referred to as optimal and non-optimal, in terms of deployment cost. The range of parameters’ values is shown in the Table 2.

In the experimental setup 2, the system’s robustness was tested with respect to the change of the swarm’s size. The number of robots was varied, and the targets’ position and quality values were preset. In the experimental setup 3, the size of the robot arena was varied to avoid specialization of the system for a specific environment. Finally, in the experimental setup 4, the performance of the system was tested with respect to different distribution of the targets of random quality values. These experimental setups were proposed in order to perform an indebt system’s performance analysis.

## 4. Results and Discussion

In this section, the results from four proposed experimental setups are presented and discussed. Each experiment was repeated 50 times.

### 4.1. Experimental Setup 1

**Figure 2.**
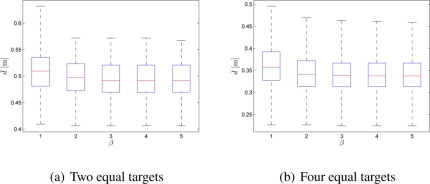
Box-plot comparison shows the average distance crossed by a robot: **(a)** 40 robots, 2 equal targets, *α* = 1; **(b)** 40 robots, 4 equal targets, *α* = 1. Each box-plot comprises observations ranging from the first to the third quartile. The median is indicated by a horizontal bar, dividing the box into the upper and lower part. The whiskers extend to the farthest data points that are within 1.5 times the interquartile range. Outliers are shown with a plus symbol. The values were obtained from 50 experiments.

It can be noticed from Table 1 that, in the Experimental Setup 1, experiments were performed with 40 robots in the fix-sized arena. Two different experiments, for 2 and 4 equal targets, were run. Control parameter *α* was set to its initial non-optimal value *α* = 1, while the control parameter *β* that biases the allocation towards the closer targets was varied. The experimental results show that when *β* was increased, the system’s performance improved with respect to the robots’ deployment cost (see Figure 2). This was measured as the median value of the distance crossed by all the robots.

### 4.2. Experimental Setup 2

**Figure 3.**
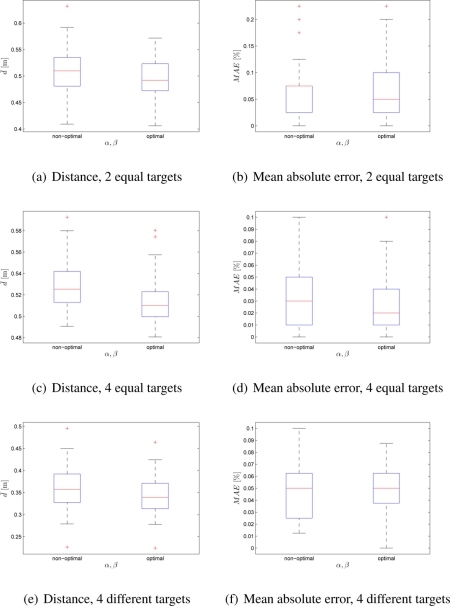
Box-plot comparison of average distance and MAE for 40 robots; *α* = *β* = 1 and *α* = 2.65, *β* = 2.55. Each box-plot comprises observations ranging from the first to the third quartile. The median is indicated by a horizontal bar, dividing the box into the upper and lower part. The whiskers extend to the farthest data points that are within 1.5 times the interquartile range. Outliers are shown with a plus symbol. The values were obtained from 50 experiments.

**Figure 4.**
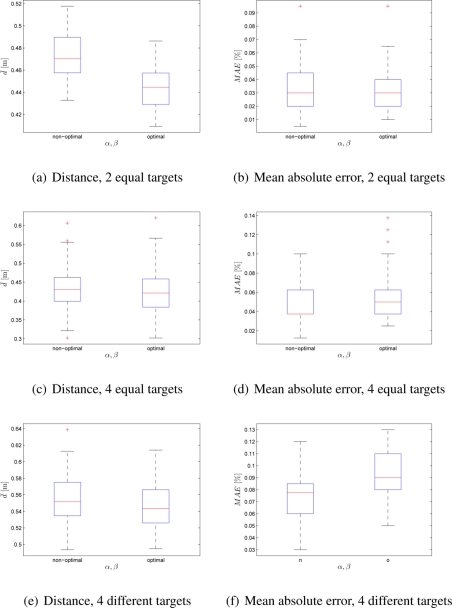
Box-plot comparison of average distance and MAE for 100 robots; *α* = *β* = 1 and *α* = 2.65, *β* = 2.55. Each box-plot comprises observations ranging from the first to the third quartile. The median is indicated by a horizontal bar, dividing the box into the upper and lower part. The whiskers extend to the farthest data points that are within 1.5 times the interquartile range. Outliers are shown with a plus symbol. The values were obtained from 50 experiments.

The Experimental Setup 2 was proposed to test the swarm’s performance when the number of robots (40 and 100) and the number of targets (2 and 4) were changed. The targets’ associated quality values were set to *q*_1_ = *q*_2_ = 0.5 and *q*_1_ = *q*_2_ = *q*_3_ = *q*_4_ = 0.25 for 2 and 4 targets, respectively. Additional experiment was performed with 4 targets that had different, but predefined, associated qualities *q*_1_ = 0.1, *q*_2_ = 0.2, *q*_3_ = 0.3, *q*_4_ = 0.4. In order to measure the swarm’s performance, median distance value and mean absolute robot distribution error were used. The experimental results for non-optimal (*α* = *β* = 1) and optimal (*α* = 2.65, *β* = 2.55) set of values are shown for 40-robot and 100-robot size swarm in Figures 3 and 4, respectively. It can be noticed that with the optimal set of control parameters swarm obtains more efficient distribution at a lower deployment cost. Only in case of 100 robots in a search of 4 targets, with equal or different qualities, the deployment cost was decreased at the expense of a higher distribution error.

### 4.3. Experimental Setup 3

**Figure 5.**
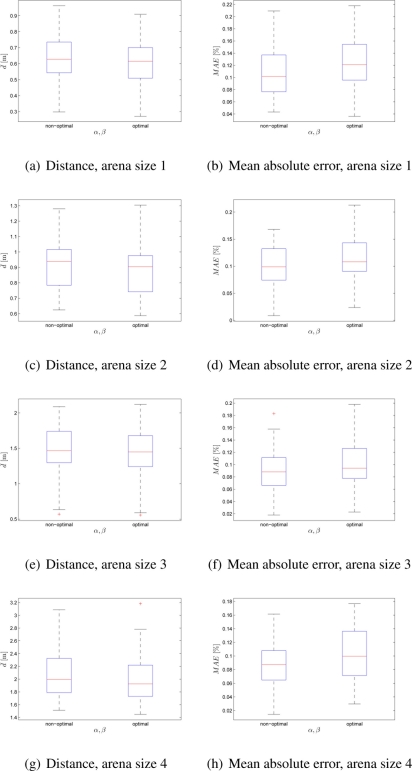
Box-plot comparison for 100 robots and 4 different randomly distributed targets; *α* = *β* = 1 and *α* = 2.65, *β* = 2.55. Each box-plot comprises observations ranging from the first to the third quartile. The median is indicated by a horizontal bar, dividing the box into the upper and lower part. The whiskers extend to the farthest data points that are within 1.5 times the interquartile range. Outliers are shown with a plus symbol. The values were obtained from 50 experiments.

This experimental setup tests swarm’s performance in case of a random distribution of the targets in the arena. Four arenas that differ in size were used (see Table 2). The scenario involved 100 robots in the search for 4 different targets with predefined quality values *q*_1_ = 0.1, *q*_2_ = 0.2, *q*_3_ = 0.3, *q*_4_ = 0.4. It can be noticed from the Figure 5 that in all the experiments the optimal control parameter values improved the performance of the swarm of robots with respect to the deployment cost. This was achieved at the expense of a higher distribution error.

### 4.4. Experimental Setup 4

**Figure 6.**
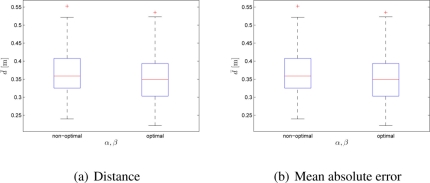
Box-plot comparison for 100 robots and 4 random-valued, randomly distributed targets; *α* = *β* = 1 and *α* = 2.65, *β* = 2.55: **(a)** distance; **(b)** MAE. Each box-plot comprises observations ranging from the first to the third quartile. The median is indicated by a horizontal bar, dividing the box into the upper and lower part. The whiskers extend to the farthest data points that are within 1.5 times the interquartile range. Outliers are shown with a plus symbol. The values were obtained from 50 experiments.

The final experiments test the swarms adaptability when the targets’ location and targets’ associated qualities are randomly chosen. The scenario considers the case of 100-robot swarm in search for 4 targets. The arena used in these experiments has 2.25 m × 3.1875 m dimension. As it can be noticed in the Figure 6, for the optimal set of control parameters’ values the performance of the swarm of robots improved with respect to both deployment cost and distribution error.

## 5. Conclusions

In this work, a swarm of robots was studied as a distributed sensors network used in search for targets within a simulated robot arena. The Distributed Bees Algorithm (DBA), which was previously proposed by the authors, was applied for distributed target allocation. This paper proposed a method for tuning the DBA’s control parameters’ values in order to achieve more efficient target allocation with respect to robots’ deployment cost. The control parameters values were optimised by means of a genetic algorithm. The improved performance was in some cases obtained at a cost of increased robots’ distribution error. Nevertheless, the proposed method allows robots to adapt their behaviour in scenarios where the resources for the robots’ deployment are limited. Taking into account large numbers of robots that can be found in a swarm, even a small improvement in a single robot’s performance can result in a significantly higher efficiency of the swarm as whole.

## Figures and Tables

**Table 1.** Parameters describing Experimental Setup 1.

	Arena 1	Arena 2
*α*	1	1
*ß*	1, 2, 3, 4, 5	1, 2, 3, 4, 5
Area dimensions [*m*^2^]	1.5 × 2.125	1.5 × 2.125
Number of robots	40	40
Experiment duration [time steps]	400	400
Time step duration [*s*]	0.1	0.1
Initial area radius [*m*]	0.4	0.4
Number of targets	2	4
Target radius [*m*]	0.09	0.09
Target 1 location (*x*_1_, *y*_1_) [*m*]	(−0.45, 0.75)	(−0.45, 0.75)
Target 2 location (*x*_2_, *y*_2_) [*m*]	(0.45, −0.75)	(0.45, −0.75)
Target 3 location (*x*_3_, *y*_3_) [*m*]	N/A	(−0.45, −0.75)
Target 4 location (*x*_4_, *y*_4_) [*m*]	N/A	(0.45, 0.75)
Target 1 quality (*q*_1_)	0.5	0.25
Target 2 quality (*q*_2_)	0.5	0.25
Target 3 quality (*q*_3_)	N/A	0.25
Target 4 quality (*q*_4_)	N/A	0.25

**Table 2.** Parameters describing experimental setups 2, 3, and 4.

Parameter	Values range
*α*	1, 2.65
*ß*	1, 2.55
Area dimensions [*m*^2^]	2.25 × 3.1875, 3.0 × 4.25, 4.5 × 6.375, 6.0 × 8.5
Number of robots	40, 100
Experiment duration [time steps]	100, 200, 300
Time step duration [*s*]	0.1
Initial area radius [*m*]	0.4, 0.5
Number of targets	2, 4
Target radius [*m*]	0.09
Targets location (*x*, *y*) [*m*]	fixed, random
Targets qualities (*q*)	fixed, random

* Selection of proposed parameters values is made for each experimental setup.
